# The Immediate Effects of Lavender Aromatherapy Massage versus Massage in Work Stress, Burnout, and HRV Parameters: A Randomized Controlled Trial

**DOI:** 10.1155/2020/8830083

**Published:** 2020-11-23

**Authors:** Chiu-Yen Wu, Hsiu-Fang Lee, C. W. Chang, Hui-Chu Chiang, Yu-Hsia Tsai, Hsueh-Erh Liu

**Affiliations:** ^1^Department of Cosmetic Science, Chang Gung University of Science and Technology, Taoyuan, Taiwan; ^2^Graduate Institute of Clinical Medical Sciences, College of Medicine, Chang Gung University, Taoyuan, Taiwan; ^3^Department of Nursing, Gung Memorial Hospital, Linkou, Taoyuan, Taiwan; ^4^Department of Nursing, Chang Gung University of Science and Technology, Taoyuan, Taiwan; ^5^School of Nursing, College of Medicine, Chang Gung University, Taoyuan, Taiwan; ^6^Division of Paediatric Endocrinology and Genetics, Department of Paediatrics, Chang-Gung Memorial Hospital, Taipei, Taiwan; ^7^School of Nursing, National Taipei University of Nursing and Health Sciences, Taipei, Taiwan; ^8^Department of Cardiovascular Medicine, Chang Gung Memorial Hospital, Linkou, Taoyuan, Taiwan; ^9^Department of Rheumatology, Chang Gung Memorial Hospital, Linkou, Taoyuan, Taiwan; ^10^Department of Nursing, College of Nursing, Chang Gung University of Science and Technology, Taoyuan, Taiwan

## Abstract

**Background:**

Occupational stress is a common issue faced by workers in every discipline. Complementary and alternative medicine (CAM) therapies, such as aromatherapy massage or massage, have antistress effects in the literature. The purpose of this randomized clinical trial with triple blinds is to evaluate the immediate effects of lavender aromatherapy massage for improving work stress, burnout, and HRV parameters of female employees in a university.

**Methods:**

A total of 53 subjects in experimental group whereas 57 subjects in control group completed interventions and measurement and led to power of 0.98. Inferential statistics, as independent *t*-test, paired *t*-test, and Chi-Square test, were performed to verify the expected relationships.

**Results:**

The present study found that subjects in experimental group reported a lower role stress and less inclined to type A personality trait after aromatherapy massage with lavender. For control group, only less inclined to type A personality trait was reported after receiving massage. For burnout, a significant lower personal burnout and work-related burnout were reported after aromatherapy massage whereas only increased client-related burnout was reported in control group. For HRV, both the experimental and control groups reported higher SDNN and RMSSD in time domain after intervention. Contradictory HRT and PSI in time domain were significantly lower after intervention. In frequency domain of HRV, both groups reported significantly higher value in VLF and HF. In addition, the experimental group reported significantly higher value in TP and LF after intervention.

**Conclusions:**

Both the lavender aromatherapy massage and massage did show immediate effect on different dimensions of work stress, burnout, and HRV. These two interventions can be applied as routine leisure activities by personal preference to reduce stresses occurring in work environment.

## 1. Introduction

People working in “person-to-person” system are easy to develop emotional burden caused by the interactions with clients, such as bank workers [1] and doctors [2]. Similar situations exist in health care workers worldwide. For clinical nurses specifically, work stresses have been identified as a “risk category” that have negative impact on their well-being and quality of patient care [3]. Nursing faculty has additional stressors than nurses who work in the acute and long-term care clinical environments. Nursing academia has been identified as a toxic work environment with many organizational stressors by a mixed-methods systematic review [4]. These occupational stresses affect the learning outcomes of nursing students, such as academic performance and welfare [5]. In addition, increased education and years of experiences have been found as predictors for intent to remain work as faculty in undergraduate and graduate nursing program [6]. Therefore, the issue of work stresses in nursing educational system is also an important issue to explore.

The interventions for reducing occupational stress in health care workers has been categorized as person-directed interventions; person–work interface interventions; and organizational interventions. The results of a systematic review show that person-directed interventions can reduce stress, burnout, and anxiety, whereas the person–work interface interventions can reduce burnout only. And the organizational interventions can reduce stress symptoms and general symptoms. However, only limited evidence found a small reduction in stress levels [7]. Recently, another review of 22 systematic reviews and meta-analyses categorizes these interventions into individual-focused, structural or organizational, and combined interventions. This review shows that all these interventions can reduce the burnout of physicians and nurses [2].

Among the various interventions reducing occupational stress, evidences of antistress effects produced by complementary and alternative medicine (CAM) therapies have been adequately established by a systematic review and meta-analysis [8]. Aromatherapy, as one of CAM, is a simple, convenient and noninvasive method of stress relief. It has been identified as a strategy of stress reduction by nurses [9].

Lavender aromatherapy and massage have been identified as the most effective treatment options for short-term treatment of anxiety [10] and with significantly increased heart rate variability (HRV) in females [11, 12]. Specifically, aromatherapy or massage can reduce the pressure of work stress on nurses. However, due to the limited numbers of studies, the review does not sufficiently prove that aromatherapy, massage, and aromatherapy massage are effectively reducing job-related stress of nurses [13]. Further investigation is needed.

Identifying stress can be challenging whereas self-response to questionnaires is commonly used in general. Decreased cardiac vagal tone, as indexed by heart rate variability (HRV), has been identified as associated with stress at work in employees [14–16]. Persistent job strain lowers the time-domain parameters in heart rate variability (HRV) of healthy nurses [17]. Using workers in high-tech company as subjects, the “work over-commitment” dimension of occupational burnout are associated with some parameters of HRV [18].

HRV can be analyzed by time- and frequency-domain methods. After lavender inhalation, significant decrease in mean heart rate (HR) and increases in standard deviation of the normal-to-normal (SDNN) intervals, square root of the mean squared differences of successive NN intervals (RMSDD), and high frequency (HF) were found [11]. Therefore, HRV can be suggested as an objective method to evaluate the levels of occupational stresses or burnout and efficiency of stress-reducing interventions [18].

The purpose of this randomized clinical trial (RCT) is to evaluate the immediate effects of lavender aromatherapy massage on work stress, burnout, and HRV parameters of female employees in a university where nursing faculties are the majority. We hypothesized that lavender aromatherapy massage could affect these indicators immediately.

## 2. Materials and Methods

### 2.1. Methods

This is a randomized clinical trial (RCT) design with triple blinds and was conducted in a university where nursing students are the majority. Subjects were recruited from that university by advertisement on the bulletin board.

### 2.2. Samples

**Figure 1:**
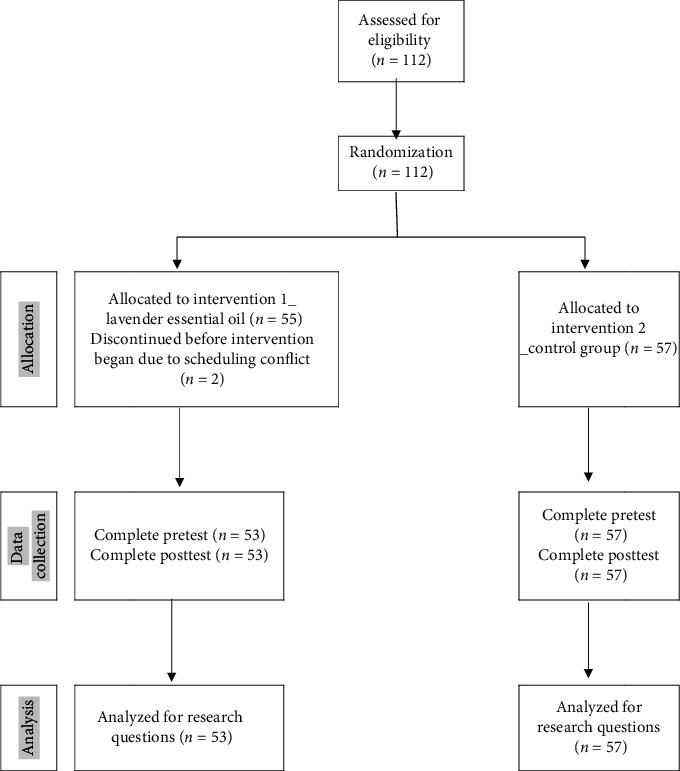
Consolidated standards of reporting trials (CONSORT) participant flow diagram.

Estimated sample size was 102 subjects while power = 0.80 was selected under the settings of middle effect size (*d* = 0.5). The inclusion criteria of subjects were (1) female; (2) age > 20 years; (3) employed more than 1 year at university; (4) conscious clear; (5) no asthma history, no allergy to essential oils, and no pregnancy; (6) have 1 hour free from being interrupted; and (7) willing to participate in this study. Those who had the history of CVA, PAOD, SCI, carotid atherosclerosis, or involuntary body movement were excluded. A total of 112 subjects were recruited; however, only 110 completed this study (Figure 1). The achieved power was 0.98, indicating a good power for this data set (effect size = 0.80, 5% for type-I error, 2-tailed).

### 2.3. Setting and Procedure

Interventions were conducted in a center with air conditioning at 24-25°C. After subjects signed informed consent, they were grouped into the experimental and control groups by drawing a lottery labeling 1 or 2. The one got sticker label 1 received massage with oil in bottle label 1 whereas the one got sticker label 2 received massage with oil in bottle label 2. Both groups of subjects received HRV measures and completed the questionnaires before and after interventions. After data analysis completed, we revealed the contents of each label and explained the results as well. Bottle label 1 contained a mixture of 5% lavender essential oil (*n* = 53), excluded 2 subjects due to the scheduling conflict before intervention started. Material in bottle label 2 was sweet almond oil (*n* = 57). The company of Taiwan branch, Potpurella, Germany, prepared these two types of aroma oil, sealed in the same brown battle, and labelled them as 1 or 2 under request of researchers.

### 2.4. Intervention: Aromatherapy Massage

A protocol with 40-minute aromatherapy massage was established by professional aromatherapy massage therapists. This intervention was conducted by individuals with basic license of aromatherapy and completed protocol training conducted by one of the research groups. The participants removed all accessories and electronics and relaxed in the center; then, they received HRV measure and completed pretest questionnaires. The same procedure of aromatherapy massage was conducted for each group except the types of oil. Finally, subjects received HRV measurement and completed questionnaires again as posttest.

### 2.5. Outcomes Measure

Three instruments were selected to measure the outcomes in the present study, which were job stressor scale (JSS), Occupational Burnout Inventory (OBI), and Heart Rate Variability Analyzer (HRV).

#### 2.5.1. Job Stressor Scale (JSS)

The primary outcome of this study was work stress. It was measured by job stressor scale (JSS). JSS is designed by Kung [19] with 55-item on 5-point Likert scale, ranging from “totally disagree = 1” to “totally agree = 5.” JSS includes 4 subscales. “The characteristics of work subscale” dealt with autonomy, completeness, variability, importance, and feedback of work; “Role stress subscale” dealt with role conflict, role ambiguity, and role overload; and the “interpersonal relationship subscale” dealt with the relationship with bosses, colleagues, subordinates, and organizations. The last subscale is “type A personality trait”; higher score indicates prone to type A personality [19]. The range of Cronbach's alpha was 0.70–0.89 in the present study.

#### 2.5.2. Occupational Burnout Inventory (OBI)

Burnout was the secondary outcome in the present study. Based on the Copenhagen Burnout Inventory (CBI) and Effort-Reward Imbalance model (ERI), OBI has been developed to measure the burden in workplace [20]. It is a self-administered 21-item questionnaire with 5-point Likert scale. Exploratory factor analysis shows that OBI has a four-subscale structure, which are personal burnout, work-related burnout, client-related burnout, and overcommitment to work. The Cronbach alpha coefficients for these four subscales are all above 0.84 in the original study whereas the range of Cronbach's alpha is 0.83–0.91 in the present study.

#### 2.5.3. Heart Rate Variability Analyzer (HRV)

The Heart Rate Variability Analyzer (HRV) was selected as objective measurement to evaluate the results of interventions in the present study. HRV has been frequently used as a physiological parameter to estimate autonomic activity. The APG Heart Rater SA-3000P (Tokyo Iken, Inagi, Tokyo) was selected to measure the HRV of subjects at pre- and postintervention. HRV is analyzed by time domain and frequency domain. The time domain measures the normal-to-normal R-R interval (NN), standard deviation of all normal-to-normal intervals (SDNN), and Root Mean Square of the differences in successive NNs (RMSSD), which can be considered as indices of cardiac parasympathetic activity. The frequency domain measures the total power (TP), the very-low-frequency power (VLF: 0.00–0.04 Hz), the low-frequency power (LF: 0.04–0.015 Hz), and the high-frequency power (HF: 0.15–0.40 Hz). Overall, VLF and HF represent parasympathetic activity whereas LF represents both sympathetic and parasympathetic activity. Meanwhile, the LF/HF ratio is considered to reflect sympathovagal balance [21–23].

#### 2.5.4. Personal Information Questionnaire

Another self-design questionnaire was selected to collect personal information of the subjects, such as age, education, marital status, work position, medical history, and habits of smoking or drinking.

### 2.6. Data Analysis

All data was managed by IBM SPSS Statistics 26. Descriptive statistics included mean, standard deviation, frequency, and percentage whereas the inferential statistics included independent *t*-test, paired *t*-test, and Chi-Square test that planned to verify the expected relationships. A two-tailed *p* < 0.05 was considered as statistical significance.

### 2.7. Ethical Considerations

This study was been approved by the Chang Gung Medical Foundation Institutional Review Board (IRB 104-9876C). All participants provided verbal and written informed consent prior to their participation.

## 3. Results

### 3.1. Personal Characteristics and Comparison between Groups

All personal characters are listed in Table 1. The subjects in experimental group were characterized as middle aged (42.53 ± 7.89 years); higher educated (77.4% > graduate school); married (52.8%); worked as faculty (39.6%); alcohol occasional drinker (52.8%); like coffee drinking (84.9%); and had no medical history (69.8%).

The characters of control group were middle aged (44.49 ± 5.89 years); higher educated (80.7% > graduate school); married (73.7%); worked as faculty (50.9%); no alcohol (50.9%) but like coffee drinking (86%); and had no medical history (77.2%). The comparison between these two groups showed that control group had a higher ratio of married subjects (*X*^2^ = 5.16, *p* = 0.023).

### 3.2. Work Stress (JSS) and Comparison between Groups

The results of mean scores of each subscale of JSS and comparisons between pretest and posttest within each group and comparison between these two groups at pretests and posttests, respectively, are listed in Table 2.

Paired *t*-tests of self-comparison between pre- and posttests in experimental group found that there was a significant difference in “role stress” (*t* = 2.862, *p* = 0.007) and “type A personality trait” subscales (*t* = 2.118, *p* = 0.041). It indicated a lower role stress and less inclined to type A personality trait after receiving aromatherapy massage. For control group, the only significant difference existed in “type A personality trait” subscale (*t* = 2.775, *p* = 0.008). It indicated a less inclined to type A personality trait after receiving massage. In addition, comparisons between control and experimental groups showed that no significant differences existed in each subscale of JSS at pretest and posttest, respectively (*p* > 0.05; Table 2).

### 3.3. Burnout (OBI) and Comparison between Groups

The results of comparisons with OBI between pretest and posttest within each group and comparison between these two groups at pre- and posttest are also listed in Table 2. For self-comparison between pre- and posttests in experimental group, paired *t*-tests found that there were significant differences in “personal burnout” (*t* = 3.996, *p* ≤ 0.001) and “work-related burnout” subscales (*t* = 3.292, *p* = 0.002). It indicated a significant lower personal burden and work-related burnout after aromatherapy massage.

For control group, the only significant difference existed in “client-related burnout” subscale (*t* = −3.336, *p* = 0.002). It indicated that control group reported increased client-related burnout after receiving massage. Meanwhile, comparisons between these two groups showed that no significant difference existed in each subscale of OBI both at pretest and posttest, respectively (*p* > 0.05; Table 2).

### 3.4. HRV and Comparison between Groups

The results of HRV, both in time domain and frequency domain, comparisons between pretest and posttest within each group and comparisons between these two groups at pre- and posttest are shown in Table 3.

For experimental group, paired *t*-tests of self-comparison between pre- and posttests found that there were significant differences in all the time domains as follows: HRT (*t* = 5.854, *p* < 0.001), SDNN (*t* = −6.105, *p* < 0.001), RMSSD (*t* = −4.768, *p* < 0.001), and PSI (*t* = 4.904, *p* < 0.001). For the frequency domain, significant differences existed in almost all domains, such as TP (*t* = −4.739, *p* < 0.001), VLF (*t* = −3.726, *p* < 0.001), LF (*t* = −0.514, *p* = 0.001), and HF (*t* = −3.036, *p* = 0.004). It indicated that lower HRT and PSI and higher SDNN and RMSSD existed in time domains whereas a higher TP, VLF, LF, and HF in frequency domains were found after aromatherapy massage.

For control group, paired *t*-tests found that there were significant differences in all time domain of HRT (*t* = 5.65, *p* < 0.001), SDNN (*t* = −4.99, *p* < 0.001), RMSSD (*t* = −2.44, *p* = 0.018), and PSI (*t* = 2.54, *p* = 0.014). For the frequency domain, significant differences only existed in VLF (*t* = −2.22, *p* = 0.031) and HF (*t* = −2.44, *p* = 0.018). It indicated that lower HRT and higher SDNN, RMSSD, and PSI existed in time domains whereas higher VLF and HF in frequency domains existed after massage.

Comparison between these two groups at pretests showed that the only significant difference existed in LF/HF (*t* = −2.012, *p* = 0.047; Table 3) in time domain at pretest whereas the significant differences existed in the TP (*t* = −3.008, *p* = 0.003) and LF (*t* = −2.359, *p* = 0.020) in frequency domain of HRV after intervention. It indicated that experimental group has a higher LF/HF ratio at pretest than subjects in control group whereas a higher TP and LF were reported by experimental group after intervention when compared with the control group.

## 4. Discussion

### 4.1. Comparison of Work Stress (JSS)

The present study found that subjects in the experimental and control groups reported similar levels in each dimension of work stress before and after intervention, indicating similar results of aromatherapy massage and massage. However, subjects in experimental group reported a lower role stress and less inclined to type A personality trait after aromatherapy massage with lavender. For control group, only less inclined to type A personality trait was reported after receiving massage.

Aromatherapy with lavender essential oil was effective in reducing job stress among nurses [24]. Lavender, the one we used, has a long history of traditional use and its essential oil was found to possess a wide range of biological effects [25, 26]. The mechanism of effects might be related to the main components of lavender as linalool and linalyl acetate. These compounds have cytotoxic effect, antioxidant power [27], and analgesic and sedative properties [28]. If aromatherapy with lavender essential oil is performed along with massage, these oils can quickly be absorbed by skin and enter the bloodstream [29]. Thus, shorter onset of lavender could be observed. Our study supported these findings from literatures that lavender with massage could reduce job stress, especially the dimension of role stress in academic nursing personnel as found in the present study.

Massage had positive effect on the stress level of nurses [24, 30] and the occupational stress in staff of the emergency medical services [31]. However, no significant difference was found in the occupational stress levels of nurses following a two 12-week periods of massage [32]. The mechanism of massage is related to the stimulation of pressure receptors that enhance vagal activity and decrease cortisol levels [33, 34]. Meanwhile, massage can stimulate the central nervous system and lead to a decrease in heart rate and respiration and create a sense of calm [35]. In the present study, subjects in both the experimental and control groups reported less inclined to type A personality trait. Type A personality trait includes competitiveness, time urgency, and a tendency toward workaholism. A duration of 40-minute massage might be not long enough to reduce the perceived stress level but the sense of calm created by massage might make subjects relax and not so consistent with the characters of type A personality trait. Therefore, both groups of subjects reported less inclined to type A personality trait.

### 4.2. Comparison of Burnout (OBI)

The present study found that a significant lower personal burnout and work-related burnout after aromatherapy massage whereas only increased client-related burnout was reported in the control group.

Personal burnout is the degree of physical and psychological exhaustion experienced by the person. It can be treated as overall perception of burnout. Work-related burnout is the degree of physical and psychological exhaustion that is related to personal work [19]. However, work factors explained only a modest part of psychological distress in nurse aides [36]. Aromatherapy with lavender essential oil reduced job stress among nurses [24]. Massage therapy led participants to greater body awareness, but no impact on job demands, social interaction, or control at work for long-term care staff [37]. Therefore, the cumulative effect of aromatherapy massage with lavender essential oil and a state of rest during interventions could reduce personal burnout and work-related burnout as found in the present study, which were supported by the literature.

Client-related burnout indicated the perceived degree of exhaustion related to his/her work with clients [19]. Present study found that after massage, the control group reported increased client-related burnout which is out of our expectation. The intervention was done at the working hours during the day. Subjects need to return to work after completing the intervention. Exposure to role conflicts and high workloads can overcome the benefits of massage therapy [37]. These might be the reasons that subjects in the control group reported higher client-related burnout after intervention. For experimental group, the additive effects of massage, lavender essential oil, and a state of resting during intervention might have greater impact on the degrees of exhaustion and make no change in client-related burnout after intervention.

### 4.3. Comparison of HRV

The present study found that, for both the experimental and control groups, all subjects reported significantly higher SDNN and RMSSD in time domain of HRV after intervention. Contradictory results were found such that significantly lower HRT and PSI in time domain of HRV after intervention were reported. In frequency domain of HRV, both groups reported significantly higher value in VLF and HF. In addition, the experimental group reported significantly higher value in TP and LF after intervention.

#### 4.3.1. Time Domain of HRV

*(1) Increased SDNN and RMSSD in Time Domain*. A systematic review showed that adverse psychosocial work conditions are negatively associated with autonomic nervous system (ANS) function as indexed by HRV [15]. In addition, decreased cardiac vagal tone was associated with stress at work in employees [14]. The main components of lavender, the one we used, are linalool and linalyl acetate. These compounds stimulate the changes on the parasympathetic activity that leads to decreased heart rate and increased high frequency (HF) in HRV [11, 28, 38, 39].

Standard deviation of the normal-to-normal intervals (SDNN) is a measure of changes in heart rate, reflecting primarily parasympathetic influences. RMSSD is the root mean square of differences of successive RR intervals. Similar to SDNN, it reflects the parasympathetic influences. High job strain was associated with lower SDNN during working day [15]. After lavender inhalation for 20 minutes, SDNN and RMSSD were increased [22, 23], and increased SDNN and RMSSD was reported in the 4th and 12th weeks of aromatherapy [11]. Similar results were found in the present study indicating that aromatherapy massage with lavender and massage could reduce the level of work stress as shown in increased SDNN and RMSSD of HRV.

*(2) Decreased HRT and PSI in Time Domain*. HRT indicates the mean of heartbeat during measurement; its normal range is 60–90 bpm. The normal range of physical stress index (PSI) is 30–50; higher score indicates higher stress of the individual. Feeling high level of stress at work was strongly associated with low cardiac vagal and increased cardiac sympathetic activity at work and caused higher heart rate [16]. Decreased HRT was observed after lavender inhalation in the 4th and 12th weeks of aromatherapy [11]. The present study found that all subjects reported a lower HRT and PSI components of HRV after interventions, indicating the relaxation effect of aromatherapy with lavender and massage.

#### 4.3.2. Frequency Domain of HRV

Frequency domain measurements estimate the distribution of absolute or relative power into four frequency bands, which are VLF, HF, TP, and LF.

*(1) VLF and HF*. The very-low-frequency (VLF) activity (0.003–0.04 Hz) and high-frequency (HF) activity (0.15 to 0.40 Hz) reflect parasympathetic nervous system (PSNS) [22, 23]. HF power increased by olfactory stimulation with linalool [39] and at the 4th and 12th weeks of lavender inhalation [11]. In addition, decreased HF was found under conditions of emotional strain [40]. Results of the present study found that both the experimental and control groups reported increased HF and VLF after interventions. This indicated more relaxation and lower stress level that subjects perceived after aromatherapy with lavender massage and massage only that have been supported in the literature.

*(2) TP and LF*. Total power (TP) is the variance of the measured signal about its mean value within frequency domain in HRV which represents a mixed ANS branch [15]. Low-frequency power (LF: 0.04–0.015 Hz) component in time domain of HRV is also representing a mixed ANS branch [15]. Subjects with lower stress feeling during the work period of a day reported a higher LF power [16]. The present study found that a higher TP and LF after intervention were reported by subjects in experimental group. It indicates the lower stress effects of aromatherapy with lavender massage in TP and LF which were supported by the literature.

*(3) LF/HF Ratio*. LF/HF ratio indicated the balance of ANS. The normal range is 0.5–2.0; a low LF/HF ratio reflects parasympathetic dominance whereas a high LF/HF ratio indicates sympathetic dominance. The present study found that experimental group has a higher LF/HF ratio than subjects in control group at pretest. It showed the hyperfunction of autonomic nervous system (ANS) that the experimental group has. However, this variation did not exist after intervention, indicating a greater impact was found in experimental group.

## 5. Limitations

Several limitations existed in this study. These were as follows. (1) Female subjects only might limit the generalization of present results to all nurse educators where male one is increasing nowadays. (2) The two groups of subjects all have interventions that make pure effect of each intervention hard to differentiate. Adding another group of pure control, that is without any interventions, then the pure effect of massage or aromatherapy massage can be detected. (3) One-time and immediate evaluation of outcomes only. Longer duration of follow-up with multiple frequency of interventions is strongly suggested. (4) The analysis of HRV parameters follows the traditional ways that did not exhibit the nonlinear perspectives of HRV. Further analysis is suggested.

## 6. Conclusions

Both the lavender aromatherapy massage and massage did have immediate impact on different dimensions of work stress and burnout, but they have similar effects on parameters of HRV. These two interventions can be applied as leisure activities by personal preference or treated as routine care for nursing educators that can decrease the levels of stresses occurring in work environment.

## Figures and Tables

**Table 1:** Comparison of demographics between groups.

Variable	Control (*n* = 57)		Lavender (*n* = 53)		*t*/*X*^2^	*p*
	*N*	%	*N*	%		
Age (year)	44.49 ± 5.89	42.53 ± 7.89	1.49	0.14		

Years of work	17.55 ± 7.05	15.48 ± 7.67	1.48	0.15		

Education level					0.19	0.67
≤University	11	19.3	12	22.6		
≥Graduate school	46	80.7	41	77.4		

Marriage					5.16	0.023
Single or divorced	15	26.3	25	47.2		
Married or have a partner	42	73.7	28	52.8		

Position					1.71	0.43
Faculty	29	50.9	21	39.6		
Staff	12	21.1	16	30.2		
Others	16	28.1	16	30.2		

Drinking					0.15	0.70
Never	29	50.9	25	47.2		
Occasionally	28	49.1	28	52.8		

Coffee drinking					0.025	0.88
No	8	14.0	8	15.1		
Yes	49	86.0	45	84.9		

Past history					0.77	0.38
No	44	77.2	37	69.8		
Yes	13	22.8	16	30.2		

**Table 2:** Comparisons of outcomes from questionnaires parameters between groups.

Variables	Control group						Experimental group						Comparison between groups			
	Pretest		Posttest		Self-comparison		Pretest		Posttest		Self-comparison		Pretest		Posttest	
	Mean	SD	Mean	SD	Paired-*t*	*p*	Mean	SD	Mean	SD	Paired *t*	*p*	*t*	*p*	*t*	*p*
Job stressor																
Characteristics of work	55.12	5.79	55.11	6.59	0.030	0.976	53.45	4.82	53.77	4.77	−0.532	0.597	1.638	0.104	1.206	0.231
Role stress	62.09	8.91	61.59	8.49	1.190	0.241	65.53	9.51	63.72	8.44	2.862	0.007	−1.958	0.053	−1.127	0.263
Interpersonal relationship	24.30	4.20	23.41	3.73	0.186	0.853	23.08	4.37	22.90	4.05	−0.368	0.715	1.497	0.137	0.595	0.554
Personality traits	42.26	4.26	40.85	3.37	2.775	0.008	41.47	4.75	40.97	4.32	2.118	0.041	0.921	0.359	−0.140	0.889

OBI																
Personal burnout	289.47	104.36	271.93	91.25	1.968	0.054	314.15	82.41	276.42	97.49	3.996	<0.0010	−1.369	0.174	−0.249	0.804
Work-related burnout	231.14	85.86	221.05	79.73	1.485	0.143	257.08	87.07	229.72	81.02	3.292	0.002	−1.572	0.119	−0.565	0.573
Client-related burnout	185.96	99.33	214.47	84.90	−3.336	0.002	219.81	107.12	202.92	98.63	1.434	0.158	−1.719	0.088	0.660	0.511
Overcommitment	306.14	87.54	299.56	97.80	0.714	0.478	307.08	100.29	293.40	88.54	1.695	0.096	−0.052	0.958	0.346	0.730

**Table 3:** Comparisons of HRV parameters between groups.

Variables	Control group						Experimental group						Comparison between groups			
	Pretest		Posttest		Paired *t*	*p*	Pretest		Posttest		Paired *t*	*p*	Pretest		Posttest	
	Mean	SD	Mean	SD			Mean	SD	Mean	SD			*t*	*p*	*t*	*p*
Time domain																
HRT	81.00	9.65	75.98	9.49	5.65	<0.001	83.72	10.41	75.92	7.99	5.854	<0.001	−1.421	0.158	0.034	0.973
SDNN	30.58	16.10	38.97	17.84	−4.99	<0.001	30.34	11.07	44.65	19.94	−6.105	<0.001	0.092	0.927	−1.577	0.118
RMSSD	22.22	17.34	26.64	15.55	−2.44	0.018	21.47	12.19	30.67	15.83	−4.768	<0.001	0.260	0.795	−1.349	0.180
PSI	107.99	146.97	62.33	62.37	2.54	0.014	96.10	85.95	44.35	32.29	4.904	<0.001	0.513	0.609	1.878	0.063

Frequency domain																
TP	890.20	1475.43	1028.42	818.82	−0.67	0.506	768.62	672.02	1769.61	1655.66	−4.739	<0.001	0.549	0.584	−3.008	0.003
VLF	461.67	813.49	698.39	759.58	−2.22	0.031	397.47	408.34	990.91	1173.90	−3.726	<0.001	0.517	0.606	−1.562	0.121
LF	236.97	395.90	281.64	442.77	−1.42	0.161	228.30	205.07	538.99	683.76	−3.514	0.001	0.143	0.887	−2.359	0.020
HF	177.70	286.58	244.08	327.72	−2.44	0.018	141.43	152.37	266.60	297.31	−3.036	0.004	0.820	0.414	−0.376	0.707
LF/HF	1.57	1.08	1.83	1.81	−0.97	0.339	2.79	4.43	2.64	2.97	0.212	0.833	−2.012	0.047	−1.736	0.085

## Data Availability

Data used in this study are available upon reasonable request from the corresponding author.
